# Social network cohesion in school classes promotes prosocial behavior

**DOI:** 10.1371/journal.pone.0194656

**Published:** 2018-04-04

**Authors:** Wouter van den Bos, Eveline A. Crone, Rosa Meuwese, Berna Güroğlu

**Affiliations:** 1 Center for Adaptive Rationality, Max Planck Institute for Human Development, Berlin, Germany; 2 Department of Psychology, University of Amsterdam, Amsterdam, the Netherlands; 3 Institute of Psychology, Leiden University, Leiden, the Netherlands; Universidad de Alicante, ITALY

## Abstract

Adolescence is a key period of social development at the end of which individuals are expected to take on adult social roles. The school class, as the most salient peer group, becomes the prime environment that impacts social development during adolescence. Using social network analyses, we investigated how individual and group level features are related to prosocial behavior and social capital (generalized trust). We mapped the social networks within 22 classrooms of adolescents aged between 12 and 18 years (N = 611), and collected data on social behaviors towards peers. Our results indicate that individuals with high centrality show both higher levels of prosocial behavior and relational aggression. Importantly, greater social cohesion in the classroom was associated with (1) reduced levels of antisocial behavior towards peers and (2) increased generalized trust. These results provide novel insights in the relationship between social structure and social behavior, and stress the importance of the school environment in the development of not only intellectual but also social capital.

## Introduction

Adolescence is a transition period during which individuals become increasingly independent of their parents, and take on adult roles in the society. It is also a period of significant social re-orientation [1,2]; there is an increase in time spent with peers and decrease in time spent with parents [3], and the peer group becomes an increasingly important source of influence on adolescent behavior [4]. In other words, adolescence is a key period for social development and thus a decisive window of opportunity to build social capital, such as trust. Adolescent development has been successfully studied from a social information processing perspective [5,6], which has put an emphasis on the development of the cognitive and affective processes underlying prosocial behavior. For instance, it is well documented that during this period most individuals become more skilled and flexible in taking the perspective of others into account [7–9]. However, it has long been stressed that the social context in which these developmental changes unfold is critical to understanding the changes during this stage of life [10]^,^[11]. Yet, our knowledge of influences of the social environment on adolescent social development is still limited.

It has long been recognized that in adolescence the peer group is one of the most salient developmental ecologies [12]. In one line of work, the study of peer relations has focused on sociometric status, or how well liked individuals are in their peer group [13]. Sociometric status in a peer group -typically a school class- is based on the number of like and dislike nominations received from individuals in the group. Another related line of research within the peer relationship literature has focused on the concept of perceived popularity [13–15]. Both measures have been valuable predictors of (the development of) prosocial and antisocial behavior[16]. In a parallel line of research, finding its roots in sociology, social network analyses were used to map the social relationships within school classes based on reports of who interacts with whom. These studies categorized adolescents’ social position in the peer network as member of a smaller clique, a liaison (bridge between cliques) or an isolate (not a member of any clique) [17]. A handful of those studies investigated the relationship between these type of network measures and social behaviors in the classroom [18–20], and showed that these measures predict unique patterns of social behavior, but also resemble perceived popularity [19].

In sum, there is ample evidence that social position in a group is related to the displays of pro- and antisocial behavior during adolescence. However, measures of individual social position or clique membership mostly ignore the complexity and influence of larger social network structures. For instance, social networks typically exhibit various levels of connection density, clustering, hierarchy, and segregation [21]. These structural differences in network structure, and their potential effects on social development, are not captured by nomination counts, clique membership, or individual network position. For instance, it has long been acknowledged that bullying is a group process, that involves multiple bystanders [20]. In addition, a recent study also showed that clique structure has an impact on the display of prosocial and aggressive behaviors beyond social position [22]. However, this study did not focus on the larger social structure that the cliques are embedded in. Furthermore, none of the previous studies focused on how peer group social structure (or position) relates to the development of social behavior outside the peer group. That is, it is not clear whether social behavior displayed in the peer group is context dependent or transfers to other social settings. The main goal of the current study is thus to extend previous work by using social network metrics to address these questions.

First, we used social network analyses to quantify group levels metrics of social cohesion within each peer group. Although social cohesion plays a central role in sociological theories of social capital [23,24], its relation with individual levels of pro- and antisocial behaviors is not well understood. Here we investigated how social cohesion in the classroom relates to behavior towards people in- and outside the classroom. We expected that increased social cohesion would be associated with increased levels of prosocial behaviors and decreased levels of antisocial behaviors. Furthermore, increased social network cohesion has been theoretically [24] and analytically [25,26] associated with increased trust, but to our knowledge never empirically. We therefore hypothesized that increased social cohesion would be associated with increased levels of generalized trust (trust is general/unfamiliar others). Trust in (unfamiliar) others is an important indicator of social capital and it has been associated with increased community engagement and higher rates of economic growth [27,28].

Second, social network analyses enable us to conduct fine-grained analyses of individuals’ position in the group (i.e., measures of centrality). Some centrality measures (e.g. eigenvector centrality) are partly overlapping with more traditional measures of perceived popularity [29], and there we expected that increased centrality would be associated with increased levels of both prosocial and antisocial behavior. However, other centrality measures (e.g. betweenness and closeness, see methods for more detail) capture unique network related positional information and we expected them to provide novel insights in the relation between position and social behavior. For instance, in a study with young adults, Brañas-Garza and colleagues have shown that centrality measures were positively correlated with altruistic behavior in a Dictator Game [30].

To address these issues we collected data from 611 adolescents in 22 different classes within one school (see also[31]). We used peer nomination data to construct the social networks that allowed us to measure both individual position and social cohesion, and investigated their relationship to social behavior. Behavior within the peer group was based on peer nominations of individuals’ prosocial (e.g., helping) and antisocial (e.g. bullying) behavior. To investigate prosocial attitudes towards general others we used an online version of the trust game [32].

## Results

### Social cohesion and social behavior

**Fig 1:**
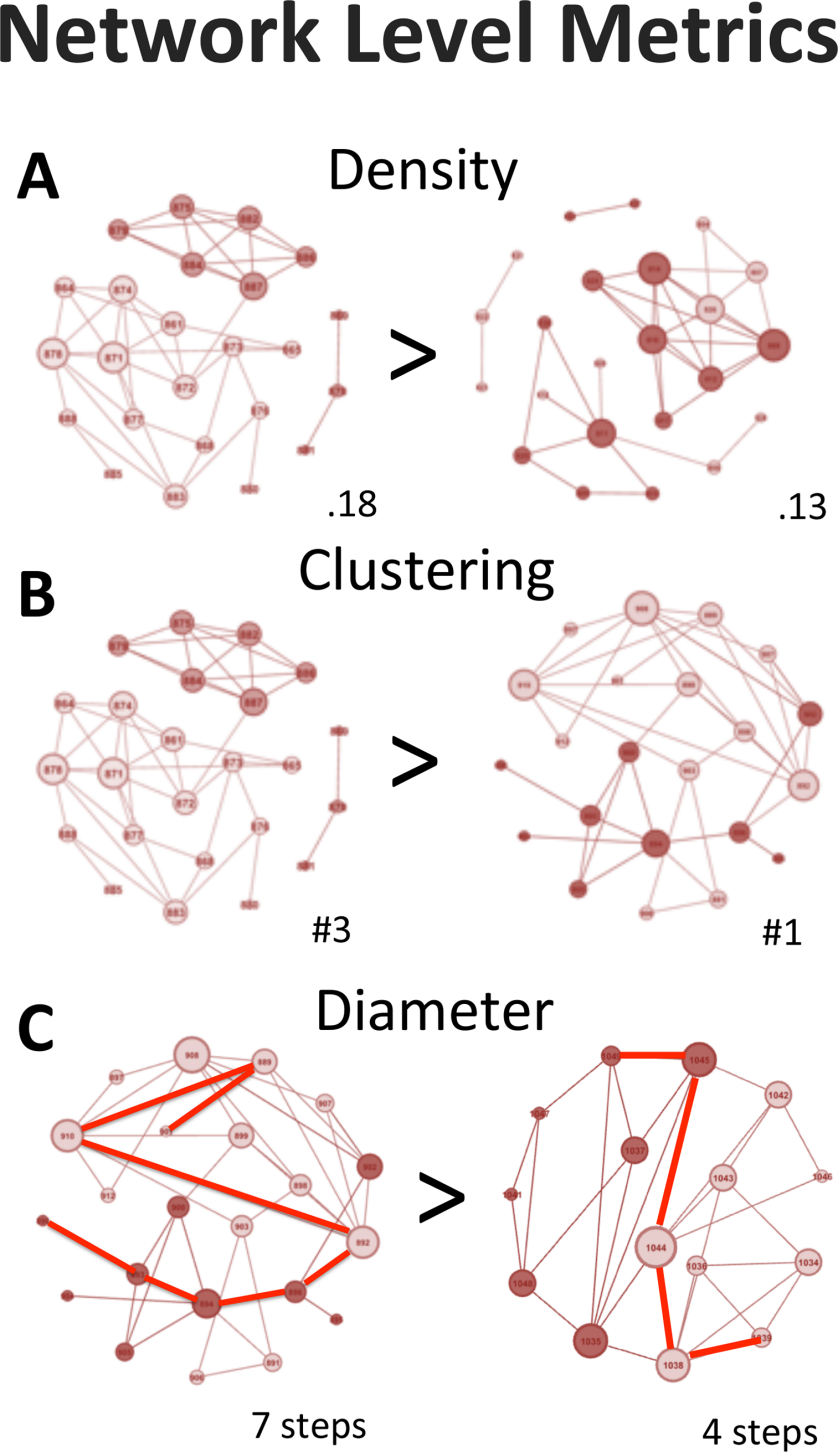
Network metrics. Illustrations of the different network metric used in our analyses based on representative classrooms from the current dataset. Each node (indicated by a circle) is a group member. The size of the nodes is associated with the total number of connections it has, and the color indicates gender (light = female). The panels present examples of the different social cohesion measures. (A) there are two networks that differ in network density. (B) shows a network that consists of 3 clusters and one network that has only one big cluster. (C) the path that is highlighted is (one of) the longest path(s) between two nodes in a network.

Based on the ‘most like’ peer nominations we constructed an undirected graph, where a connection between nodes was created when there was a reciprocal nomination (see Fig 1 for a representative network). Note that this method differs from other social network studies that used reciprocal friendship nominations (cf. [33]). However, a large meta-analysis examining children’s reciprocal friendship nominations has shown that friends are significantly more likely to provide mutual liking nominations compared to non-friend dyads, unilateral friends, and acquaintances [34]. Therefore, to stay as close as possible to previous studies that have used friendship nominations as basis for network construction (e.g. [35]) we have used reciprocal nominations only. In addition, previous studies have shown that reciprocal relationships are good predictors of social behaviors in adolescence [36]. The ‘like’ nominations also allowed us to embed our results in other sociometrics measures that have traditionally been used in developmental studies such as preference and popularity (see Supplementary Materials S1 Text for more details).

A wide range of social attributes and behaviors was assessed based on peer nominations. For the analyses of social behavior in the classroom we focused on composite scores that describe four different types of behavior (cf. [37,38]): 1) prosocial behavior (cooperating and helping), 2) antisocial behavior (getting in physical fight, start arguments and bullying), 3) relational aggression (ignoring, excluding and gossiping), and 4) victimization (being excluded, ignored, bullied and gossiped about; see Methods for more details).

The first set of analyses focused on how the overall level of social cohesion, as measured with social network metrics, was associated with frequency of different types of social behavior in the classroom. These analyses focused on three measures of social cohesion: density, diameter, and cluster count (for representative networks see Fig 1). The *density* measure gives an indication of how many relations between individual exists relative to all possible relationships that could exist (total number edges / total possible number of edges). In this context a high density network is one where there are many reciprocal peer nominations. *Diameter* measure gives an indication of the distances in a network (see Fig 1). In a high diameter network there are individuals who almost never interact. On the other hand, low diameter indicates high cohesion; everybody knows each other well or is a “friend of a friend”. Finally, the number of *clusters* is inversely related with cohesion: the more subgroups there are the less the overall cohesion of the group. Based on previous modeling work [25,26] we expected that increased social cohesion would be associated with increased levels of prosocial behavior both in- and outside the classroom.

Frequency of social behaviors within the group was entered as the dependent variable in a multiple mixed model regression with social cohesion measures, age, and gender ratio as independent variables (see Table 1). The results highlight that group level measures were only associated with the level of antisocial behavior in the group. More specifically, increased amounts of antisocial behavior were associated with lower social cohesion, as measured by lower network density (*β* = -.884, *p* < .02), and larger group sizes (*β* = .771, *p* < .02).

**Table 1:** Group level: Results of multiple logistic regressions with aggregate social behavior as dependent variables.

	Prosocial	Antisocial	Relational	Victimization
Diameter	-0.123(-0.409, 0.162)	-0.351(-0.557, -0.144)	0.074(-0.865, 0.482)	0.345(-0.006, 0.695)
Clusters	-0.293(-0.645, 0.06)	-0.078 (-0.418, 0.261)	-0.028 (-0.388, 0.332)	0.211(-0.222, 0.643)
Density	-0.511(-0.978, -0.044)	-0.884(-1.208, -0.56)*	0.132(-0.345, 0.609)	0.077(-0.497, 0.65)
Gender ratio	-0.38(-0.681, -0.08)	0.528 (0.291, 0.764)	-0.385(-0.692, -0.078)	0.061(-0.308, 0.43)
Age	-0.194 (-0.429, 0.041)	-0.213(-0.399, -0.027)*	-0.047(-0.287, 0.193)	-0.177(-0.465, 0.112)
Group size	-0.558(-0.928, -0.188)	0.771(0.491, 1.05)*	-0.199 (-0.576, 0.179)	0.062(-0.392, 0.516)

All regression models included age, group size, gender ratio (1 = all boys), and individual frequencies of social behaviors as independent variables. Individual unstandardized β’s are reported (95% confidence interval in parentheses) Note.

* p < .05 (FDR corrected)

**Fig 2:**
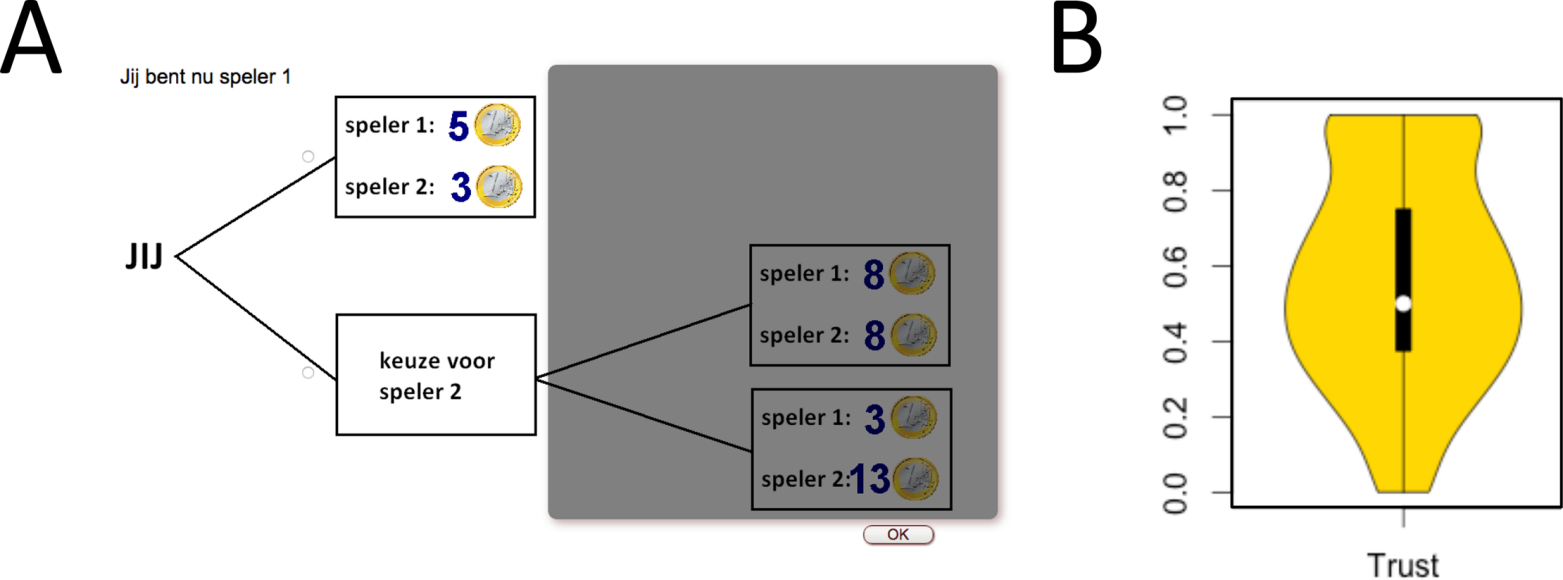
The Trust game. (A) When the participant was the first player he or she could decide to either trust or not trust the other person. The possible outcomes for trusting where shaded to indicate the role of the participant but still clearly visible to understand the possible consequences. **(**B) Distributions of percentages of trust choices, white circle is the population mean.

Next, we considered the effect of social network structure on generalized trust. Generalized trust is the trust displayed in interactions with unfamiliar others and is not based upon previous interactions or information acquired about that individual. We used an online version of the binary choice Trust Game [32,39,40] to assess generalized trust (see Fig 2). Before the participants played the Trust Game they were instructed that these games would have real (monetary) consequences for both players and that one of the games would be randomly picked at the end of the experiment to determine their pay-off. The identity of both players was kept completely anonymous; the only information that was revealed was that the other person was of the same age and gender as the participant (see supplement for more details; S1 Text).

The percentage of trust choices was entered as the dependent variable in a multiple mixed model regression analysis with the network positions, age, and gender as independent variables. Consistent with previous findings [41], we found that boys show more trust than girls (*β* = -.149, *p* < .005). More importantly, overall levels of social cohesion were associated with levels of trust (see Table 2). That is, increased number of clusters and larger diameter, both indicating lower social cohesion, were associated with lower levels of trust (*β* = -.302, *p* < .001 and *β* = -.239, *p* < .02, respectively).

**Table 2:** Group level: Results of multiple logistic regression analysis with generalized trust as dependent variable.

	Trust
Diameter	**-.239**(-.463, -.015)*
Clusters	**-.302**(-.470, -.135)***
Density	-.222(-.461, .016)
Gender ratio	-.017(-.296, .263)
Age	.234(.084, .383)**
Group size	-.074(-.007, .012)
Constant	.326(.209, .445)***

All regression models included age, group size, gender ratio (1 = all boys), and individual frequencies of social behaviors as independent variables. Individual unstandardized *β*’s are reported (95% confidence interval in parentheses) Note.

* p < .05

** p < .01

*** p < .001 (FDR corrected)

### Social position and social behavior

**Fig 3:**
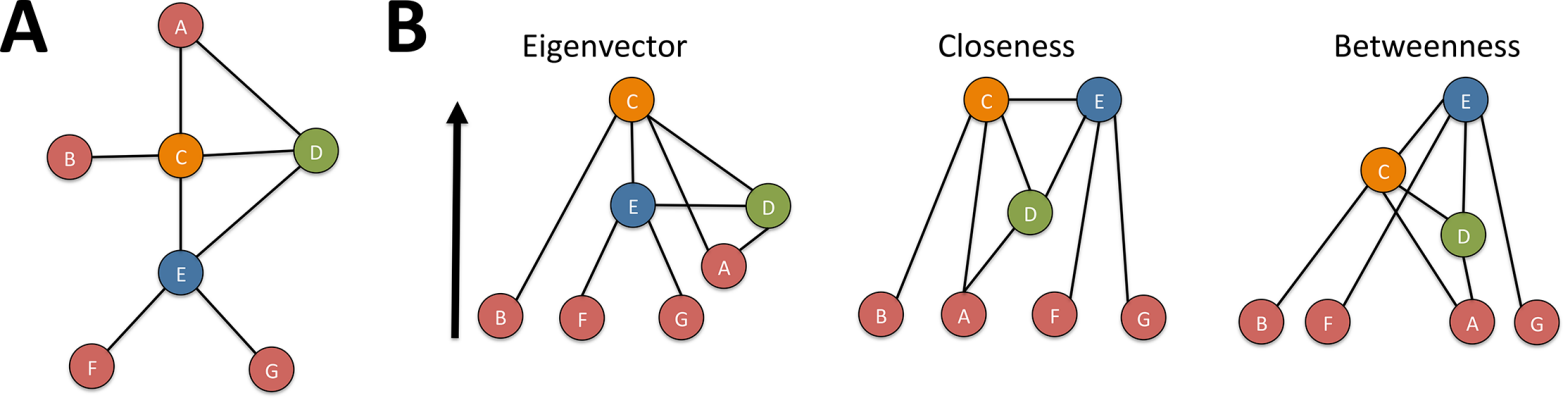
Individual level metrics. (A) an example of a hypothetical social network illustrating the individual level metrics. The letters label individuals in the network.(B) the same network is restructured in a hierarchy such that the node with the highest relevant centrality measure is on top (following the arrow). For example, node C has the highest Eigenvector centrality, but node E had the highest betweenness centrality.

Finally, we used individual social network metrics as measures of individual differences in position in the peer group. The individual network metrics reflect different aspects of influence and importance within the network. We focused on three standard social network metrics (eigenvector-, betweenness- and closeness centrality) to see how individual position within the social network is related to specific types of behavior (see Fig 3). *Eigenvector* centrality is an indicator of the importance of individuals in the network that takes into account the number of connections an individual has and the quality of the nodes it is connected to (in turn determined by number of connections). In other words, high eigenvector indicates a high level of influence rather than simply having many connections. Second, *betweenness* centrality gives an indication of how important a person is for the transmission of information through different parts of the network. Third, *closeness* centrality indicates whether someone is in the center or the fringe of the network. Someone with high closeness centrality is in close connection with those at the top and well as the bottom of the hierarchy (see S7 Table for correlation between centrality measures). Given that eigenvector centrality is conceptually related to the perceived popularity [29], we expected that they would be associated with increased levels of both prosocial and antisocial behavior [14]. However, given that the other metrics also capture unique network related to variance, we expected them to provide novel insights in the relation between individual position in the peer group and social behavior. Note that we did not include degree centrality (number of connections) in our analyses given that it is conceptually, as well as statistically (see S7 Table), very hard to distinguish from eigenvector centrality, and measures of popularity that are also based on simple connection counts. Because the eigenvector centrality measure provides more information about an individual’s place within the network, we chose this metric over degree centrality.

The first set of analyses focused on the how centrality measures were associated with peer-reported individual social behavior in the classroom. The same four types of social behaviors as used above were entered as dependent variables in separate multiple mixed model regression analyses with the individual network positions, age, and gender as independent variables. Even though the correlation between in the independent variables were low (see S7 Table), or nonsignificant, we have checked whether our regression models suffered from multicollinearity by calculating variance inflation factors (VIFs). These analyses revealed that none of the models suffered from multicollinearity, all VIFs < 3, and thus allowed us to confidently estimate the unique relation of the different centrality measures with the dependent variable of social behavior. This allowed us to find the unique variance in the individual network measures that explain the social behavior of interest.

In line with previous findings, high eigenvector centrality was associated with both high levels of prosocial behavior and high levels of relational aggression (*β* = 2.54, *p* < .001 and *β* = .129, *p* < .008, respectivelyö see Table 3). Also, in line with expectations, those individuals at the top of the social network were the least likely to be victimized (*β* = -.325, *p* < .001). In sum, the eigenvector centrality measures behave similar to perceived popularity measures (but see S1 Text), firmly embedding our edge measure (reciprocal likes) into the existing literature. Betweenness centrality, however, was related to lower levels of relational aggression (*β* = -.09, *p* < .02). Closeness centrality did not show any significant relations with classroom behavior. Finally, in line with previous work, girls showed more prosocial behavior (*β* = -1.93, *p* < .001), as well as more relational aggression (*β* = -.203, *p* < .001), than boys; boys displayed more antisocial behavior (*β* = .253, *p* < .001). Finally, we performed exploratory analyses where we constructed interaction terms with gender or age; however, in none of the models, these terms turned out to be significant (all p’s >.4) . In the last set of analyses we also examined the relation between individual network position and trust; the results showed that none of the individual network metrics were associated with generalized trust (see S4 Table).

**Table 3:** Results of multiple logistic regressions with social behaviors as dependent variables.

	Prosocial	Antisocial	Relational	Victimization
Eigenvector	**.254**(.166, .342)***	.043(-.036, .121)	**.129**(.048, .210)**	**-.325**(-.394, -.256)***
Betweenness	.034(-.048, .116)	-.056(-.130, .012)	**-.090**(-.166, -.015)*	-.045(-.109, .020)
Closeness	-.007(-.093, .080)	.010(-.067, .088)	-.007(-.087, .073)	.012(-.057, .080)
Age	-.014(-.095, .066)	.008(-.064, .080)	.007(-.067, .081)	-.011(-.075, .053)
Gender	**-.193**(-.272, -.114)***	.**253**(.181, .324)***	**-.203**(-.276, -.130)***	-.045(-.107, .017)
Constant	.053(-.026, .131)	-.022(-.092, .048)	-.013(-.086, .059)	-.101(-.163, -.038)**

Logistic regression models included age, gender (1 = male, 0 = female) and individual network level metrics as independent variables. Individual unstandardized β‘s are reported (95% confidence interval in parentheses) Note.

* p < .05

** p < .01

*** p < .001 (FDR corrected).

## Discussion

The main purpose of this study was to investigate the relation between social structure and adolescents’ social behavior by utilizing social network analyses to identify individual and peer group characteristics. The social network analyses allowed us to separate effects with respect to the analytic level where they occurred: 1) the group level with measures describing social cohesion, and 2) the individual level with factors associated with each person’s position in a network. Our novel approach enabled us to extend previous findings in three ways. First, we were able to investigate the relation between group level features and social behavior. Second, we were able to investigate to what extend the link between network characteristics and social behavior generalizes towards people outside the network and contributes to trust as an index of social capital. Third, the social network analyses allowed a more fine-grained analysis of the relationship between social position and social behavior. Taken together, our results provide evidence for the influence of social position *and* structure on behavior in- *and* outside the classroom. In the discussion we will first address our conclusions regarding social behavior within the classroom followed by behavior outside the classroom.

Consistent with previous studies we found that individuals who are central in the network structure show both increased prosocial behavior, as well as increased relational aggression [14,18,19]. It is important to note that individuals with high eigenvector centrality—individuals of high importance in the network—did not necessarily engage in antisocial behavior (such as fighting), which might lead to being disliked, but in relational aggression, which is more likely to indicate manipulative behavior in interpersonal relationships and thus increase one’s position in the group. These findings underline the conclusions from previous studies that taking a central role in the network is not only achieved by being nice [14], and validate that the centrality measures in this network pick up relevant social relations (for further comparisons with traditional measures see S1 Text). It is important to note also that those with a high betweenness centrality—individuals who have reciprocal likes with individuals from different parts of the network- stand out by showing less relational aggression. This novel finding suggests that it is possible to occupy a significant role in the network without relying on relational aggression (even refraining from it). Indeed, our analyses also revealed that betweenness centrality predicted how much peers liked you, but not how popular you are (See Supplemental Results).

Importantly, we also found that social cohesion was associated with reduced levels of antisocial behavior in the classroom. Additionally, we found that that group size is negatively correlated with cohesion. The larger the group, the less dense the network is, and the more clusters it has (see S3 Table). Group size is a unique variable given that this is one of the few exogenously induced variables, which is, in principle, controllable. Although our data is cross-sectional, it suggests two possible causal pathways that may lead to this association. First, increased group size may result in reduced density and more clusters, which in turn leads to more antisocial behavior and a reduction in generalized trust. Such a pathway between group size, social cohesion, and antisocial behavior may explain mixed reports about the relation between class size and bullying behavior. For instance, O’Moore and colleagues reported that smaller classes show less bullying [42], whereas others have failed to find this relationship [43,44]. Thus, class size may not be directly related to the amount of antisocial behavior, but antisocial behavior may increase in larger classrooms due to changes in their social structure. Indeed, several studies have shown that antisocial children are more popular in hierarchical as compared to egalitarian classrooms [19,45,46]. However, a second possibility is that class size directly leads to an increase in antisocial behavior and/or reduced trust, which in turn might have a detrimental effect on social cohesion. This hypothesized pathway is supported by studies that show that increased group size can indeed lead to reductions in interpersonal trust [47,48]. Regardless of whether the exact causal structure is correct, our results underline that it is crucial to understand the determinants of the social structure in the classroom in order to contribute to interventions programs aimed at reducing antisocial behavior and promoting prosocial behavior. In addition, both pathways suggest that decreasing class size may be an important tool to increase social cohesion and trust, and reduce antisocial behavior.

Finally, we investigated the impact of the social network structure on generalized trust. On the individual level, none of the network metrics predicted the level of trust in unknown others, but, in line with our hypothesis, we found that higher levels of social cohesion were associated with increased generalized trust. Such an effect is also in line with sociological and economic theories about the role of social structure on social capital [23,24]. For instance, recent social network analyses have shown how network cohesion could contribute to the emergence and sustainability of cooperative behavior [49,50]. These studies show that more effective communication, and therefore social control (e.g., punishment), in cohesive networks can lead to more cooperation and trust [49,50]. In addition, the theory of social capital suggests that the trust, or social capital, built up in one context is transferable to another [27]. This notion of transfer is partly supported by our finding that there was a trending relationship between social cohesion and the amount of reported prosocial behavior. To conclude, this implies that the classroom may be a valuable source of social capital [27], regardless of the social position of an individual. We already pointed out that class size may play an important role in increasing cohesion, and thus be a focal point for policy, but earlier research also indicated that there might be an important role for the schools and teachers [51]. For instance, one study showed that promoting the classroom as a unit in school might have positive impact on stability of social relations and scholarly engagement of pupils [52]. Our work suggests that such interventions may also contribute to increasing social capital.

The current study does not come without limitations. The most obvious limitation is the cross-sectional design of our study, whereby causal interpretations (directionality) of underlying mechanisms remain speculative. Longitudinal studies are necessary to identify developmental trajectories of social behavior that can help crystallize the reciprocal influence of social structure and social behavior. Interestingly, one longitudinal study suggests that in early to mid-adolescence the social networks are rather stable, at least over the period of one year [17]. One of the most interesting starting points would therefore be to examine the formation of a new class at the beginning of secondary school. This would allow us to grasp how social behavior contributes to the formation of the network structure and later how that structure starts to co-shape social behavior. It is also important to point out that the network structure is a function of the elicitation method that we used [53]. The reciprocal “likes” measure we have used here is close to the often used “friend” nominations, but may deviate in subtle ways. On a related note, as is the case with typical friend nominations our measures do not allow for weighted networks, which might seem unrealistic given that not all relationships are equal [54,55]. Here we have related our measure to relevant sociometric measures used in the traditional peer relationships literature [14,16], but future studies are needed to demonstrate how these measures are related to, and predict prosocial behavior. Such studies could also explore additional centrality measures, given that the current study only focused on a subset of them (such as Katz centrality or PageRank [56]).

The current findings provide a promising starting point for studying the relation of social network struccture to the presence of prosocial behavior in adolescence. With regards to possible policy implications it is interesting to highlight our findings regarding group size. The main focus of discussion about classroom sizes has been the teacher to pupil ratio and the effect of group size on the academic performance of the pupils. Regardless which stance is taken in that debate, the current results suggest that there might be another argument for limiting group sizes: social capital [57]. It is important for future studies to examine how these network metrics relate to and reflect the impact of specific school programs that are aimed at increasing prosocial behavior and class cohesion, such as anti-bullying programs (e.g., good behavior game [58]) and democracy education [59]. The network metrics may serve as short-term indicators of the success of these interventions. Crucially, this line of research should next include the mapping of these measures onto real-world prosocial behaviors such as volunteering.

## Methods

### Participants

We recruited 22 classes from a single school in the Netherlands resulting in a total of 611 (321, female) participants, with a mean age of 14.8 (*SD* = 1.49) years. The classes ranged from the first grade (12–13 years) to the sixth grade (17–18 years) of high school. Two classes were excluded for further analyses because they both consisted of two mixed classes that changed in composition over time and both differed significantly in size (N = 55 and N = 60 respectively); the mean class size after exclusion was 27.7 (*SD* = 7.7) and total N = 496.

Most participants were of Dutch origin (84%), as were most of their parents (70%); other participants were of various origins around the globe (both European and non-European). Participants were recruited and data was collected in March and April 2011 from a local secondary school in an affluent suburb of in of the larger cities the Netherlands.

### Measures

#### Peer nominations & social behavior

A wide range of social attributes and behaviors was assessed based on peer nominations. These included questions about likeability (i.e., ‘Who do you like the most?’ and ‘Who do you like the least?’) and popularity (i.e., ‘Who is most popular in the class?’ and ‘Who is least popular in the class?’), as well as overt behaviors such as helping, bullying and gossiping (see Text S2 Text for the complete list of items). Participants could nominate an unlimited number of peers for each category but self-nominations were not allowed. The number of nominations of certain type of behavior a group member received was interpreted as a proxy for how often an individual displays this behavior within the peer group. For the analyses of social behavior we focused on composite scores that describe four different types of behavior (cf [37,38]); 1) prosocial behavior (cooperating and helping), 2) antisocial behavior (getting in physical fight, start arguments and bullying), 3) relational aggression (ignoring, excluding and gossiping), and 4) victimization (being excluded, ignored, gossiped about and bullied). However, for exploratory purposes we have also included item level regressions in the supplementary results (S1 Text). We have used the nominations for the item ‘who do you like most?’ to construct the social networks for each classroom. Finally, age and gender were included in the individual level models; on the group level we have included class size, mean age and gender ratio in all analyses. Note that both age and gender are the focus of another study (in preparation) and that these variables were essentially included as possible variables to see how the unique variance of the network measures is related to the behavioral outcomes. Therefore, these effects will not be further discussed here, but will be examined in a separate manuscript. The peer nomination data is available at https://osf.io/vucwf/files/

#### Social network analyses

The network structure of each group and individual node properties (eigenvector centrality, betweenness and closeness) were analyzed using the igraph package (version 1.0; http://igraph.org) for R [60], and visualized in Gephi [61]. We used three standard centrality metrics to indicate individual positions within the social network: eigenvector, betweenness and closeness centrality. Three network level metrics were used as indicators for social cohesion: density, diameter and number of clusters. Clusters within a network were determined using the clusters algorithm based on edge betweenness [62] provided by the igraph package. Further details about these metrics are described below.

#### Individual level metrics

We investigated three standard social network metrics (eigenvector-, betweenness- and closeness centrality) to see how individual position within the social network is related to specific types of behavior (see Fig 1). *Eigenvector* centrality is an indicator of importance of individuals in the network as it takes into account the number and quality of connections an individual has, where the quality of a connected node is measured by how many connections that node has (see Fig 1). In this respect, eigenvector centrality is often seen as a measure of importance. Because this measure takes the larger structure of the network into account it gives a richer account of how important someone is within the network compared to other sociometric measures based on the number of counts such as degree centrality or absolute differences (e.g. likes–dislikes). Second, *betweenness* centrality gives an indication of how important a person is for the transmission of information through the network. Betweenness is calculated by dividing the number of shortest paths between all pairs of nodes in the network that pass through the specific node divided by the total number of shortest paths in the network [63]. People with high betweenness centrality can be seen as gatekeepers between different clusters in the network. Third, *closeness* centrality indicates whether someone is in the center or the fringe of the network. Closeness is based on the sum of the shortest paths between a node and all other nodes [64]. Someone with high closeness centrality is in close connection with those at the top and well as the bottom of the hierarchy. In sum, the social network metrics combine the advantage of continuous measures as used in earlier sociometrics (e.g., popularity) with different social network categories as used in previous social network analyses.

### Experimental procedures

Informed consent was obtained from the school principal and the parents of participants. The local ethics committee of Leiden University approved the study and the experiment was performed in accordance with all relevant guidelines and regulations. All participants were tested in their own classroom as part of a larger study accompanied by four trained experimenters. The first half of the testing session consisted of questionnaires measuring different aspects of development, such as psychosocial functioning, social behavior, and peer relationships, followed by eight different economic games including the trust game used in the current study (see [31], trust game data available at https://osf.io/vucwf/files/). Finally, all participants completed a short cognitive capacity test. Each testing session lasted approximately 60 min. Only four groups had missing data of maximally one participant who happened to be absent on the day of testing.

Before the testing session started participants were encouraged to ask questions. It was emphasized that participation was voluntary and it was ensured that all data would be handled confidentially and anonymously. The first screen that was presented before the economic games provided a reminder that the participants were playing the allocation games for real money and that at the end of data collection one person within every class would be randomly chosen to receive the money he or she earned in the four games. Each coin in the game was worth €1. Two weeks after testing the experimenters returned to the schools to give one participant in each class their earnings; participants received €5 on average.

### Statistical procedures

For all individual behaviors, multiple hierarchical regression models were used in order to take into account the structure of the data (subjects nested in classrooms). The lmer function of the lme4 library in R was used to estimate the mixed models [65], with variable intercepts and fixed slopes per classroom, using parametric estimation with the default **bobyqa** algorithm for numerical optimization. Note that fixed slopes models showed superior goodness-of-fit compared to variable slope models based on the standard Bayesian model comparison indicators. Degrees of freedom and *p* values for mixed effects models were calculated with the lmerTest library in R, using Satterthwaite’s approximation [66]. Finally all reported *p*-values associated with the regression models are adjusted for multiple comparisons using false discovery rates [67].

Group level network analyses were performed to obtain the social cohesion measures (e.g. clustering, density). To examine how social cohesion was related to social behavior within the classroom we calculated for each of the social behavioral categories the total number of individuals within the classroom that were reported to show this type of behavior (e.g. prosocial behavior). This total number was transformed to a ratio by dividing it by the total number of individuals in the classroom. Next, we calculated the average level of generalized trust within a social network. Finally, given that the diameter and number of clusters are related to group size, we also included total group size in all the group level analyses. To get robust estimates of standard errors of our regression analyses, we have used the sandwich variance estimator, as implemented in the **sandwich** package for R [68].

## Supporting information

S1 Text Individual level metrics, preference & popularity. (DOCX)

S2 Text Supplemental methods. (DOCX)

S1 Table Correlation matrix for behavior in the classroom. (DOCX)

S2 Table Correlations matrix for individual attributes. (DOCX)

S3 Table Correlations matrix for group size attributes. (DOCX)

S4 Table Individual level statistics: Results of multiple logistic regressions with generalized trust as dependent variable. (DOCX)

S5 Table Results of multiple logistic regressions with individual network metrics. (DOCX)

S6 Table Classroom statistics. (DOCX)

S7 Table Correlations matrix for node attributes. (DOCX)
