# Supplementary material for: Social network cohesion in school classes promotes prosocial behavior
Source: PLoS One. 2018 Apr 4;13(4):e0194656. doi: 10.1371/journal.pone.0194656 (PMC5884510; doi:10.1371/journal.pone.0194656)
Supplement: S1 Text — (DOCX) [file pone.0194656.s001.docx]

**Text S1. Individual level metrics, preference & popularity**

Finally, to get a sense of how the reported centrality measures relate to sociometric measures often used in the peer relationship literature, we have compared them to the more traditional measures of social preference and perceived popularity. Preference was based the total like nominations by the other members of the peer group; perceived popularity was based on the total number of popular nominations. Using total number of positive nominations yielded the same results as using difference scores (e.g. total likes minus total dislikes or total popular minus total unpopular nomination). These analyses allowed us to further embed our current findings in the existing peer relationships literature.

First, replicating earlier studies [1] we found that there was a weak but significant positive correlation between sociometric and perceived popularity (r = .28, p < .01). More importantly, we found that the centrality measures are related to the traditional popularity measures (see Table 5). First, eigenvector centrality was correlated with both sociometric and perceived popularity. As expected, individuals who are more liked, and those who are perceived to be more popular take up important positions within the social network (more towards the top of the hierarchy). A similar finding, but slightly weaker, emerged for the relation between closeness centrality. Interestingly, betweenness centrality showed a positive correlation with sociometric popularity and a trending negative relation with perceived popularity. Thus, those individuals who have a key role in connecting different parts of the network are liked a lot but are not necessarily perceived to be popular and were even slightly perceived to be unpopular (trending *p* = .07). Note, that the three metrics are all associated, in different ways, with age and gender (Table 5). Overall the findings suggest that boys take up more ‘important’ network positions, and that these positions also are related to older ages (i.e. the older the more important). To conclude, the network metrics are able to capture unique variance of social preference and perceived popularity, but, consistent with previous literature [2], the levels of explained variance also suggest that centrality measures capture independent dimensions of social position.

1. Cillessen AHN, Rose AJ. Understanding Popularity in the Peer System. Curr Dir Psychol Sci. 2005;14: 102–105. doi:10.1111/j.0963-7214.2005.00343.x

2. Gest SD, Graham-Bermann SA, Hartup WW. Peer Experience: Common and Unique Features of Number of Friendships, Social Network Centrality, and Sociometric Status. Soc Dev. 2001;10: 23–40. doi:10.1111/1467-9507.00146
