# Supplementary material for: Social network cohesion in school classes promotes prosocial behavior
Source: PLoS One. 2018 Apr 4;13(4):e0194656. doi: 10.1371/journal.pone.0194656 (PMC5884510; doi:10.1371/journal.pone.0194656)
Supplement: S2 Text — (DOCX) [file pone.0194656.s002.docx]

**S2 Text. Supplemental methods**

| 1. Which children from the class do you like the most? |
| --- |
| 1. Which children from the class do you like the least? |
| 1. Which children from the class are the most popular? |
| 1. Which children from the class are the least popular? |
| 1. Which children from the class are good cooperators? (P) |
| 1. Which children from the class often help others? (P) |
| 1. Which children from the class ignore others? (R) |
| 1. Which children from the class are ignored by others? (V) |
| 1. Which children from the class are shy? |
| 1. Which children from the class get in physical fights with others? (A) |
| 1. Which children from the class gossip about other children? (R) |
| 1. Which children from the class are gossiped about? (V) |
| 1. Which children from the class exclude others? (R) |
| 1. Which children from the class are excluded? (V) |
| 1. Which children from the class get into arguments with others? (A) |
| 1. Which children from the class bully others? (A) |
| 1. Which children from the class are being bullied? (V)   P = prosocial, R = relational aggression, A = anti social behavior, V = victimized |

**Table S1 Correlation matrix for behavior in the classroom.**

|  | Help  others | Ignore | Being ignored | Shy | Physical fight | gossip | gossiped about | Exclude | Being Excluded | Argument | Bully | Being Bullied |
| --- | --- | --- | --- | --- | --- | --- | --- | --- | --- | --- | --- | --- |
| Help others | 1 |  |  |  |  |  |  |  |  |  |  |  |
| Ignore | -.09 | 1 |  |  |  |  |  |  |  |  |  |  |
| Being ignored | -.09 | .09 | 1 |  |  |  |  |  |  |  |  |  |
| Shy | -.10 | -.21 | .26 | 1 |  |  |  |  |  |  |  |  |
| Physical fight | -.27 | .28 | **-.50**** | .00 | 1 |  |  |  |  |  |  |  |
| Gossip | .43 | .13 | .20 | .18 | -.10 | 1 |  |  |  |  |  |  |
| Gossiped about | .00 | **-.49**** | -.21 | .28 | -.05 | .31 | 1 |  |  |  |  |  |
| Exclude | .11 | .30 | -.44 | -.34 | .38 | -.03 | -.27 | 1 |  |  |  |  |
| Being Excluded | -.05 | -.14 | .46 | .22 | -.17 | -.09 | -.15 | **-.63**** | 1 |  |  |  |
| Argument | .31 | **.58**** | -.09 | **-.51**** | .25 | .21 | -.21 | -.02 | .04 | 1 |  |  |
| Bully | .16 | .40 | -.20 | -.31 | **.52**** | .32 | -.19 | .39 | -.35 | .41 | 1 |  |
| Being Bullied | .07 | .15 | .36 | .17 | -.08 | .00 | -.22 | **-.54**** | **.71***** | .28 | -.24 | 1 |

*Cells represent Pearson correlation (r) between measures, *p<.05; **p<.01;* ***p<.001

**Table S2**

|  | | | | |  |  |  |
| --- | --- | --- | --- | --- | --- | --- | --- |
|  | | | | |  |  |  |
|  | helping | shy | bullies | bullied | | gossips | gossiped |
|  |  |  |  |  | |  |  |
| Closeness | -.011  (-.106, .084) | .019  (-.064, .101) | .005  (-.084, .094) | .012  (-.062, .086) | | -.012  (-.095, .071) | .001  (-.079, .081) |
| Betweenness | .029  (-.061, .119) | -.039  (-.116, .039) | -.067  (-.151, .017) | -.037  (-.107, .033) | | -.057  (-.136, .021) | **-.079***  (-.155, -.003) |
| Eigenvector | **.223*****  (.127, .319) | **-.378*****  (-.461, -.295) | .059  (-.031, .149) | **-.327*****  (-.402, -.252) | | **.208*****  (.124, .292) | **-.240*****  (-.321, -.158) |
| Age | -.002  (-.090, .086) | -.038  (-.114, .038) | .007  (-.075, .090) | -.018  (-.087, .050) | | .002  (-.074, .079) | .003  (-.071, .077) |
| Gender | **-.246*****  (-.333, -.159) | **-.152*****  (-.227, -.077) | .**250*****  (.169, .331) | .027  (-.040, .095) | | **-.454*****  (-.529, -.378) | **-.200*****  (-.274, -.127) |
| Constant | .044  (-.042, .129) | -.066  (-.141, .008) | -.010  (-.090, .070) | -.103**  (-.169, -.036) | | -.003  (-.078, .071) | -.100**  (-.172, -.028) |
|  | | | | |  |  |  |

|  | | | | |  |  |
| --- | --- | --- | --- | --- | --- | --- |
|  | | | | |  |  |
|  | argument | physical | ignored | excluded | | cooperative |
|  |  |  |  |  | |  |
| Closeness | .029  (-.061, .118) | -.002  (-.086, .082) | .028  (-.050, .106) | .001  (-.076, .078) | | -.002  (-.094, .090) |
| Betweenness | -.044  (-.129, .041) | -.056  (-.136, .023) | -.025  (-.098, .047) | -.036  (-.106, .034) | | .039  (-.048, .126) |
| Eigenvector | -.024  (-.115, .067) | **.093*** (.008, .178) | **-.377*****  (-.455, -.299) | **-.356*****  (-.431, -.282) | | **.286***** (.193, .379) |
| Age | -.002  (-.085, .081) | .018  (-.060, .096) | -.014  (-.086, .059) | -.015  (-.089, .058) | | -.026  (-.111, .059) |
| Gender | **.090*** (.008, .172) | **.418***** (.341, .495) | -.013  (-.083, .057) | .007  (-.061, .074) | | **-.140****  (-.224, -.056) |
| Constant | -.046  (-.127, .035) | -.010  (-.086, .065) | -.093*  (-.164, -.022) | -.106**  (-.177, -.034) | | .061  (-.021, .144) |
| *Note:* **p<.05; **p<.01; ***p<.001* | | | | |  |  |

**Table S3 Correlations matrix for group size attributes.**

|  | Density | Diameter | | Clusters | | Gender ratio | | Age | | | Group size |
| --- | --- | --- | --- | --- | --- | --- | --- | --- | --- | --- | --- |
| Density | 1.00 | |  | |  | |  | |  |  | |
| Diameter | 0.28 | | 1.00 | |  | |  | |  |  | |
| Clusters | -0.55** | | -0.49* | | 1.00 | |  | |  |  | |
| Gender ratio | 0.61** | | -0.01 | | -0.20 | | 1.00 | |  |  | |
| Age | 0.05 | | 0.16 | | -0.24 | | -0.02 | | 1.00 |  | |
| Group size | -0.62** | | -0.01 | | 0.57* | | -0.35 | | -0.19 | 1.00 | |

*Cell represents Pearson correlation (r) between measures, *p<.05; **p<.01;* ***p<.001

**Table S4. Individual level statistics: Results of multiple logistic regressions with generalized trust as dependent variable**

|  | Trust |
| --- | --- |
|  |  |
| Eigenvector | -.069  (-.171, .033) |
| Betweenness | -.003  (-.098, .092) |
| Closeness | .081  (-.026, .188) |
| Age | .095  (-.010, .199) |
| Gender | .145** (.052, .237) |
| Constant | -.002  (-.104, .100) |
| Logistic regression models included age, gender (1= male, 0=female) and individual network level metrics as independent variables. unstandardized β’s are reported. Note. * p < .05; ** p < .01. | |

***Individual level metrics, preference & popularity***

Finally, to get a sense of how the reported centrality measures relate to sociometric measures often used in the peer relationship literature, we have compared them to the more traditional measures of social preference and perceived popularity. Preference was based the total like nominations by the other members of the peer group; perceived popularity was based on the total number of popular nominations. Using total number of positive nominations yielded the same results as using difference scores (e.g. total likes minus total dislikes or total popular minus total unpopular nomination). These analyses allowed us to further embed our current findings in the existing peer relationships literature.

First, replicating earlier studies ^14^ we found that there was a weak but significant positive correlation between sociometric and perceived popularity (r = .28, p < .01). More importantly, we found that the centrality measures are related to the traditional popularity measures (see Table 5). First, eigenvector centrality was correlated with both sociometric and perceived popularity. As expected, individuals who are more liked, and those who are perceived to be more popular take up important positions within the social network (more towards the top of the hierarchy). A similar finding, but slightly weaker, emerged for the relation between closeness centrality. Interestingly, betweenness centrality showed a positive correlation with sociometric popularity and a trending negative relation with perceived popularity. Thus, those individuals who have a key role in connecting different parts of the network are liked a lot but are not necessarily perceived to be popular and were even slightly perceived to be unpopular (trending *p* = .07). Note, that the three metrics are all associated, in different ways, with age and gender (Table 5). Overall the findings suggest that boys take up more ‘important’ network positions, and that these positions also are related to older ages (i.e. the older the more important). To conclude, the network metrics are able to capture unique variance of social preference and perceived popularity, but, consistent with previous literature ^61^, the levels of explained variance also suggest that centrality measures capture independent dimensions of social position.

**Table S5. Results of multiple logistic regressions with individual network metrics**

|  | | | |
| --- | --- | --- | --- |
|  | Eigenvector | Betweenness | Closeness |
|  | (1) | (2) | (3) |
| Preference | **.440****  (.367, .512) | **.249****  (.161, .337) | .**107****  (.083, .131) |
| Popularity | .**281****  (.210, .351) | **-.080**  (-.166, .006) | .**033****  (.009, .056) |
| Age | .043  (-.092, .177) | **.157****  (.040, .274) | **.090***  (.015, .164) |
| Gender | **.108****  (.039, .177) | -.022  (-.106, .062) | **.029***  (.006, .052) |
| Constant | .078  (-.071, .228) | -.040  (-.158, .078) | .273  (-.185, .731) |
| *Note:* | | *p<.05; p<.01;* p<.001 | |
