# Supplementary material for: Social network cohesion in school classes promotes prosocial behavior
Source: PLoS One. 2018 Apr 4;13(4):e0194656. doi: 10.1371/journal.pone.0194656 (PMC5884510; doi:10.1371/journal.pone.0194656)
Supplement: S1 Table — (DOCX) [file pone.0194656.s003.docx]

**Table S1 Correlation matrix for behavior in the classroom.**

|  | Help  others | Ignore | Being ignored | Shy | Physical fight | gossip | gossiped about | Exclude | Being Excluded | Argument | Bully | Being Bullied |
| --- | --- | --- | --- | --- | --- | --- | --- | --- | --- | --- | --- | --- |
| Help others | 1 |  |  |  |  |  |  |  |  |  |  |  |
| Ignore | -.09 | 1 |  |  |  |  |  |  |  |  |  |  |
| Being ignored | -.09 | .09 | 1 |  |  |  |  |  |  |  |  |  |
| Shy | -.10 | -.21 | .26 | 1 |  |  |  |  |  |  |  |  |
| Physical fight | -.27 | .28 | **-.50**** | .00 | 1 |  |  |  |  |  |  |  |
| Gossip | .43 | .13 | .20 | .18 | -.10 | 1 |  |  |  |  |  |  |
| Gossiped about | .00 | **-.49**** | -.21 | .28 | -.05 | .31 | 1 |  |  |  |  |  |
| Exclude | .11 | .30 | -.44 | -.34 | .38 | -.03 | -.27 | 1 |  |  |  |  |
| Being Excluded | -.05 | -.14 | .46 | .22 | -.17 | -.09 | -.15 | **-.63**** | 1 |  |  |  |
| Argument | .31 | **.58**** | -.09 | **-.51**** | .25 | .21 | -.21 | -.02 | .04 | 1 |  |  |
| Bully | .16 | .40 | -.20 | -.31 | **.52**** | .32 | -.19 | .39 | -.35 | .41 | 1 |  |
| Being Bullied | .07 | .15 | .36 | .17 | -.08 | .00 | -.22 | **-.54**** | **.71***** | .28 | -.24 | 1 |

*Cells represent Pearson correlation (r) between measures, *p<.05; **p<.01;* ***p<.001
