# Supplementary material for: Social network cohesion in school classes promotes prosocial behavior
Source: PLoS One. 2018 Apr 4;13(4):e0194656. doi: 10.1371/journal.pone.0194656 (PMC5884510; doi:10.1371/journal.pone.0194656)
Supplement: S2 Table — (DOCX) [file pone.0194656.s004.docx]

**Table S2 Correlations matrix for individual attributes**

|  | | | | |  |  |  |
| --- | --- | --- | --- | --- | --- | --- | --- |
|  | | | | |  |  |  |
|  | helping | shy | bullies | bullied | | gossips | gossiped |
|  |  |  |  |  | |  |  |
| Closeness | -.011  (-.106, .084) | .019  (-.064, .101) | .005  (-.084, .094) | .012  (-.062, .086) | | -.012  (-.095, .071) | .001  (-.079, .081) |
| Betweenness | .029  (-.061, .119) | -.039  (-.116, .039) | -.067  (-.151, .017) | -.037  (-.107, .033) | | -.057  (-.136, .021) | **-.079***  (-.155, -.003) |
| Eigenvector | **.223*****  (.127, .319) | **-.378*****  (-.461, -.295) | .059  (-.031, .149) | **-.327*****  (-.402, -.252) | | **.208*****  (.124, .292) | **-.240*****  (-.321, -.158) |
| Age | -.002  (-.090, .086) | -.038  (-.114, .038) | .007  (-.075, .090) | -.018  (-.087, .050) | | .002  (-.074, .079) | .003  (-.071, .077) |
| Gender | **-.246*****  (-.333, -.159) | **-.152*****  (-.227, -.077) | .**250*****  (.169, .331) | .027  (-.040, .095) | | **-.454*****  (-.529, -.378) | **-.200*****  (-.274, -.127) |
| Constant | .044  (-.042, .129) | -.066  (-.141, .008) | -.010  (-.090, .070) | -.103**  (-.169, -.036) | | -.003  (-.078, .071) | -.100**  (-.172, -.028) |
|  | | | | |  |  |  |

|  | | | | |  |  |
| --- | --- | --- | --- | --- | --- | --- |
|  | | | | |  |  |
|  | argument | physical | ignored | excluded | | cooperative |
|  |  |  |  |  | |  |
| Closeness | .029  (-.061, .118) | -.002  (-.086, .082) | .028  (-.050, .106) | .001  (-.076, .078) | | -.002  (-.094, .090) |
| Betweenness | -.044  (-.129, .041) | -.056  (-.136, .023) | -.025  (-.098, .047) | -.036  (-.106, .034) | | .039  (-.048, .126) |
| Eigenvector | -.024  (-.115, .067) | **.093*** (.008, .178) | **-.377*****  (-.455, -.299) | **-.356*****  (-.431, -.282) | | **.286***** (.193, .379) |
| Age | -.002  (-.085, .081) | .018  (-.060, .096) | -.014  (-.086, .059) | -.015  (-.089, .058) | | -.026  (-.111, .059) |
| Gender | **.090*** (.008, .172) | **.418***** (.341, .495) | -.013  (-.083, .057) | .007  (-.061, .074) | | **-.140****  (-.224, -.056) |
| Constant | -.046  (-.127, .035) | -.010  (-.086, .065) | -.093*  (-.164, -.022) | -.106**  (-.177, -.034) | | .061  (-.021, .144) |
| *Note:* **p<.05; **p<.01; ***p<.001* | | | | |  |  |
