# Supplementary material for: Social network cohesion in school classes promotes prosocial behavior
Source: PLoS One. 2018 Apr 4;13(4):e0194656. doi: 10.1371/journal.pone.0194656 (PMC5884510; doi:10.1371/journal.pone.0194656)
Supplement: S3 Table — (DOCX) [file pone.0194656.s005.docx]

**Table S3 Correlations matrix for group size attributes.**

|  | Density | Diameter | | Clusters | | Gender ratio | | Age | | | Group size |
| --- | --- | --- | --- | --- | --- | --- | --- | --- | --- | --- | --- |
| Density | 1.00 | |  | |  | |  | |  |  | |
| Diameter | 0.28 | | 1.00 | |  | |  | |  |  | |
| Clusters | -0.55** | | -0.49* | | 1.00 | |  | |  |  | |
| Gender ratio | 0.61** | | -0.01 | | -0.20 | | 1.00 | |  |  | |
| Age | 0.05 | | 0.16 | | -0.24 | | -0.02 | | 1.00 |  | |
| Group size | -0.62** | | -0.01 | | 0.57* | | -0.35 | | -0.19 | 1.00 | |

*Cell represents Pearson correlation (r) between measures, *p<.05; **p<.01;* ***p<.001
