# Supplementary material for: Social network cohesion in school classes promotes prosocial behavior
Source: PLoS One. 2018 Apr 4;13(4):e0194656. doi: 10.1371/journal.pone.0194656 (PMC5884510; doi:10.1371/journal.pone.0194656)
Supplement: S4 Table — (DOCX) [file pone.0194656.s006.docx]

**Table S4. Individual level statistics: Results of multiple logistic regressions with generalized trust as dependent variable**

|  | Trust |
| --- | --- |
|  |  |
| Eigenvector | -.069  (-.171, .033) |
| Betweenness | -.003  (-.098, .092) |
| Closeness | .081  (-.026, .188) |
| Age | .095  (-.010, .199) |
| Gender | .145** (.052, .237) |
| Constant | -.002  (-.104, .100) |
| Logistic regression models included age, gender (1= male, 0=female) and individual network level metrics as independent variables. unstandardized β’s are reported. Note. * p < .05; ** p < .01. | |
