# Supplementary material for: Social network cohesion in school classes promotes prosocial behavior
Source: PLoS One. 2018 Apr 4;13(4):e0194656. doi: 10.1371/journal.pone.0194656 (PMC5884510; doi:10.1371/journal.pone.0194656)
Supplement: S5 Table — (DOCX) [file pone.0194656.s007.docx]

**Table S5. Results of multiple logistic regressions with individual network metrics**

|  | | | |
| --- | --- | --- | --- |
|  | Eigenvector | Betweenness | Closeness |
|  | (1) | (2) | (3) |
| Preference | **.440****  (.367, .512) | **.249****  (.161, .337) | .**107****  (.083, .131) |
| Popularity | .**281****  (.210, .351) | **-.080**  (-.166, .006) | .**033****  (.009, .056) |
| Age | .043  (-.092, .177) | **.157****  (.040, .274) | **.090***  (.015, .164) |
| Gender | **.108****  (.039, .177) | -.022  (-.106, .062) | **.029***  (.006, .052) |
| Constant | .078  (-.071, .228) | -.040  (-.158, .078) | .273  (-.185, .731) |
| *Note:* | | *p<.05; p<.01;* p<.001 | |
