# Supplementary material for: Social network cohesion in school classes promotes prosocial behavior
Source: PLoS One. 2018 Apr 4;13(4):e0194656. doi: 10.1371/journal.pone.0194656 (PMC5884510; doi:10.1371/journal.pone.0194656)
Supplement: S6 Table — (DOCX) [file pone.0194656.s008.docx]

|  | Demographics (average) | |  | Network level metrics | |  |  | Node level metrics (average) | |  |  |  | Peer nominations (average) | |  |
| --- | --- | --- | --- | --- | --- | --- | --- | --- | --- | --- | --- | --- | --- | --- | --- |
| Class | age | gender ratio |  | density | clusters | path length |  | closeness | betweenness | eigenvector | degree |  | nominations | reciprocal nominations | |
| 1 | 13.35 | 0.46 |  | 0.37 | 1.00 | 2.67 |  | 0.01 | 21.70 | 0.34 | 4.81 |  | 8.30 | 0.58 |  |
| 2 | 13.44 | 0.42 |  | 0.28 | 4.00 | 1.70 |  | 0.00 | 2.30 | 0.29 | 3.04 |  | 5.35 | 0.57 |  |
| 3 | 13.37 | 0.54 |  | 0.30 | 2.00 | 2.62 |  | 0.00 | 14.44 | 0.29 | 3.85 |  | 7.26 | 0.53 |  |
| 4 | 13.02 | 0.64 |  | 0.34 | 2.00 | 2.69 |  | 0.01 | 14.96 | 0.40 | 3.92 |  | 6.92 | 0.57 |  |
| 5 | 13.86 | 0.59 |  | 0.35 | 1.00 | 2.57 |  | 0.02 | 16.50 | 0.40 | 3.73 |  | 5.86 | 0.64 |  |
| 6 | 13.62 | 0.52 |  | 0.29 | 3.00 | 2.09 |  | 0.00 | 4.96 | 0.29 | 3.33 |  | 5.17 | 0.65 |  |
| 7 | 13.67 | 0.58 |  | 0.32 | 3.00 | 2.63 |  | 0.00 | 13.42 | 0.36 | 3.96 |  | 6.65 | 0.60 |  |
| 8 | 13.38 | 0.38 |  | 0.34 | 3.00 | 2.16 |  | 0.01 | 7.91 | 0.37 | 3.74 |  | 5.61 | 0.67 |  |
| 9 | 14.61 | 0.56 |  | 0.54 | 1.00 | 2.49 |  | 0.03 | 10.40 | 0.55 | 3.80 |  | 5.87 | 0.65 |  |
| 10 | 14.53 | 0.69 |  | 0.38 | 2.00 | 1.98 |  | 0.01 | 3.15 | 0.39 | 2.31 |  | 5.15 | 0.45 |  |
| 11 | 15.23 | 0.35 |  | 0.56 | 1.00 | 2.67 |  | 0.03 | 11.67 | 0.49 | 3.93 |  | 5.00 | 0.79 |  |
| 12 | 15.41 | 0.45 |  | 0.35 | 4.00 | 1.91 |  | 0.00 | 7.00 | 0.37 | 4.79 |  | 8.75 | 0.55 |  |
| 13 | 16.02 | 0.54 |  | 0.32 | 3.00 | 2.25 |  | 0.01 | 7.38 | 0.39 | 3.24 |  | 5.43 | 0.60 |  |
| 14 | 16.50 | 0.40 |  | 0.55 | 1.00 | 2.40 |  | 0.03 | 9.07 | 0.46 | 3.57 |  | 5.07 | 0.70 |  |
| 15 | 16.68 | 0.57 |  | 0.62 | 1.00 | 2.27 |  | 0.05 | 6.36 | 0.66 | 3.09 |  | 5.00 | 0.62 |  |
| 16 | 16.35 | 0.57 |  | 0.21 | 2.00 | 3.28 |  | 0.00 | 37.83 | 0.24 | 4.67 |  | 7.93 | 0.59 |  |
| 17 | 17.94 | 0.54 |  | 0.17 | 2.00 | 3.33 |  | 0.00 | 38.98 | 0.22 | 3.28 |  | 5.78 | 0.57 |  |
| 18 | 15.03 | 0.70 |  | 0.51 | 1.00 | 2.25 |  | 0.02 | 11.90 | 0.46 | 4.80 |  | 7.20 | 0.67 |  |
| 19 | 15.29 | 0.81 |  | 0.44 | 1.00 | 2.55 |  | 0.02 | 15.48 | 0.39 | 4.38 |  | 7.62 | 0.58 |  |
| 20 | 14.33 | 0.47 |  | 0.34 | 1.00 | 2.64 |  | 0.02 | 14.79 | 0.54 | 3.05 |  | 4.74 | 0.64 |  |
| 21 | 15.18 | 0.46 |  | 0.39 | 1.00 | 2.62 |  | 0.02 | 17.83 | 0.56 | 4.30 |  | 6.91 | 0.62 |  |

**Table S6 Classroom statistics.**

Class/group level statistics. All but the network level social network metrics represent the mean.
