# Supplementary material for: Social network cohesion in school classes promotes prosocial behavior
Source: PLoS One. 2018 Apr 4;13(4):e0194656. doi: 10.1371/journal.pone.0194656 (PMC5884510; doi:10.1371/journal.pone.0194656)
Supplement: S7 Table — (DOCX) [file pone.0194656.s009.docx]

**Table S7 Correlations matrix for node attributes.**

|  | betweenness | closeness | eigenvector | degree |
| --- | --- | --- | --- | --- |
| betweenness | 1 |  |  |  |
| closeness | 0.13* | 1 |  |  |
| eigenvector | 0.31* | 0.17* | 1 |  |
| degree | 0.56* | 0.16* | 0.82* | 1 |

*Cell represents Pearson correlation (r) between measures, **p<.001
